# Effects of *Taraxacum officinale* on Fatigue and Immunological Parameters in Mice

**DOI:** 10.3390/molecules171113253

**Published:** 2012-11-07

**Authors:** Bo-Ra Lee, Jong-Hyun Lee, Hyo-Jin An

**Affiliations:** 1 Division of Herbal Medicinal Products, Korea Food & Drug Administration (KFDA), Osong Health Technology Administration Complex, 187 Osongsaengmyeong2(i)-ro, Osong-eup, Cheongwon-gun, Chungcheongbuk-do 363-700, Korea; Email: leebora44@korea.kr; 2 College of Pharmacy, Dongduk Women’s University, 23-1 Wolgok-dong, Seongbuk-gu, Seoul 136-714, Korea; Email: naturalmed@dongduk.ac.kr; 3 Department of Pharmacology, College of Oriental Medicine, Sangji University, 83 Sangjidae-gil, Wonju-si, Gangwon-do 220-702, Korea

**Keywords:** *Taraxacum officinale*, forced swimming test, mouse peritoneal macrophage, cytokines, nitric oxide

## Abstract

In Korean herbal medicine dandelion (*Taraxacum officinale*, TO) has been used to improve energy levels and health. However, the effects of TO in experimental models remain unclear. We examined the anti-fatigue and immune-enhancing effects of TO in mice by performing a forced swimming test (FST) and *in vitro* by using peritoneal macrophages, respectively. After daily oral administration of TO, blood biochemical parameters related to fatigue were measured after the FST. FST immobility time was significantly decreased in the TO-treated group (100 mg/kg) on the tenth day. TO (10 and 100 mg/kg) treatment significantly increased glucose levels, acting as an energy source. The level of lactic dehydrogenase, which is an accurate indicator of muscle damage, tended to decline after TO administration (10 and 100 mg/kg). When TO (100 mg/kg) was orally administered to mice, blood urea nitrogen levels decreased significantly. We also examined the effect of TO on the production of cytokines and nitric oxide (NO) in mouse peritoneal macrophages. When TO was used in combination with recombinant interferon-gamma (rIFN-γ), a noticeable cooperative induction of tumor necrosis factor-alpha (TNF-α), interleukin (IL)-12p70, and IL-10 production was observed. Furthermore, in peritoneal macrophages, rIFN-γ plus TO treatment significantly increased the production of NO through inducible nitric oxide synthase (iNOS) induction. Taken together, these results suggest that TO improves fatigue-related indicators and immunological parameters in mice.

## 1. Introduction

Some plants of the genus *Taraxacum*, commonly known as dandelion, have long been used in folk medicine for the treatment of hepatic disorders and some women’s diseases, such as cancers of the breast and uterus, and as a lactation aid, choleretic, diuretic, and anti-inflammatory agent [1,2]. Thus far, the pharmacological activities of *Taraxacum* plants, particularly *Taraxacum officinale* (TO) F. Weber ex Wiggers (Asteraceae), have only been evaluated partly [3]. TO was reported to demonstrate acute anti-inflammatory activity with a protective effect against cholecystokinin-induced acute pancreatitis in rats [4]. It was also shown to induce apoptosis of human hepatoma HepG2 cells through tumor necrosis factor-α (TNF-α) and interleukin (IL)-1β secretion, and to have cytotoxic activity in the human colon colorectal adenocarcinoma cell line Caco-2 [5,6]. Furthermore, in primary cultures of rat astrocytes stimulated with lipopolysaccharide (LPS) and TNF-α-inducing substance P, TOsignificantly inhibited the production of TNF-α by inhibiting IL-1β production, indicating that TO has an anti-inflammatory effect in the central nervous system [7]. However, the immune-enhancing effect of TO is not completely understood.

The forced swimming test (FST) is a behavioral test for rodents that has been used to predict the efficacy of antidepressant treatments [8]. This test induces the development of immobility as a reflection of helplessness when subjected to an inescapable situation (a deep water tank). In this model, mice are placed in the tank for an extended period. After an initial swimming period, the animals exhibit immobility behavior, which is considered a depression-like response. Thus, FST is used to examine whether an agent has an anti-fatigue effect [9,10,11]. Blood urea nitrogen (BUN), creatine kinase (CK), and lactic dehydrogenase (LDH) are blood biochemical parameters related to fatigue. The BUN test is a routine test that is primarily used to evaluate renal function. Urea is formed in the liver as the end product of protein metabolism. During digestion, proteins are broken down into amino acids. Amino acids contain nitrogen, which is removed as NH_4_^+^ (an ammonium ion), whereas the remainder of the molecule is used to produce energy or other substances needed by cells. Therefore, an increased level of BUN indicates that the cells require more energy. CK and LDH are known to be accurate indicators of muscle damage. The normal function of CK in cells is to add a phosphate group to creatine, turning it into the high-energy molecule phosphocreatine. Phosphocreatine is used as a quick source of energy by cells [12]. Exercising muscles convert glucose (Glc) to lactate. Lactate is released into the blood and is eventually taken up by the liver. The liver converts lactate back to Glc and releases Glc into the blood. This Glc is then taken up by resting muscles, red blood cells, and other tissues. Energy for exercise is derived initially from the breakdown of glycogen, and later from circulating Glc released by the liver and from non-esterified fatty acids [13]. It is commonly known that Glc levels are decreased immediately after exercise. Albumin (Alb) is the most abundant plasma protein in blood. Protein measurements can reflect nutritional state, kidney disease, liver disease, and many other underlying conditions. Enzymes, some hormones, hemoglobin, low-density lipoprotein (LDL), fibrinogen, and immunoglobulins are some examples of proteins.

Macrophages are involved in many different processes, such as tissue remodeling during embryogenesis, wound repair, removal of damaged or senescent cells subsequent to injury or infection, hemopoiesis, and homeostasis. In addition, macrophages serve as a line of defense against microbial invasion, and recognize and kill tumor cells. Macrophages can accomplish this in a direct manner, which involves the release of products such as oxygen radicals and TNF-α that are harmful to microorganisms or cancer cells [14]. They also play an indirect role in antimicrobial and antitumor activities by secreting cytokines (e.g., IL-12) or by antigen processing and presentation, thereby regulating the immune system [15]. IL-10 is an important immunoregulatory cytokine that is produced by numerous cell types. It is an important inhibitor of many aspects of the inflammatory response. In LPS-activated macrophages, IL-10 has strong inhibitory effects on the production of pro-inflammatory cytokines (IL-1β, IL-6, and TNF-α) and chemokines (IL-8 and macrophage inflammatory peptide-1α), generation of nitric oxide (NO), and upregulation of surface antigen expression (MHC class II, CD80, and CD86) [16]. 

NO is a highly reactive molecule that is produced from the guanidino nitrogen of arginine by NO synthase (NOS). During the past decade, NO has received attention as a potent macrophage-derived effector molecule against various bacteria, parasites, and tumors. Evidence of tumor cell cytostasis and cytotoxicity was demonstrated in macrophage tumor cell co-cultures in which cytokine and/or LPS-stimulated macrophages suppressed the metabolic functioning of co-cultured tumor cells [17]. Various immune-enhancing substances regulate the production of NO.

In the present study, we examined the anti-immobility effects of TO during FST. After FST, the effects of TO on biochemical parameters related to fatigue were evaluated. In addition, we examined the regulatory effects of TO on immunological parameters, such as TNF-α, IL-12p70, and IL-10, and NO production in mouse peritoneal macrophages.

## 2. Results and Discussion

### 2.1. Effect of TO on the Immobility Time

**Figure 1:**
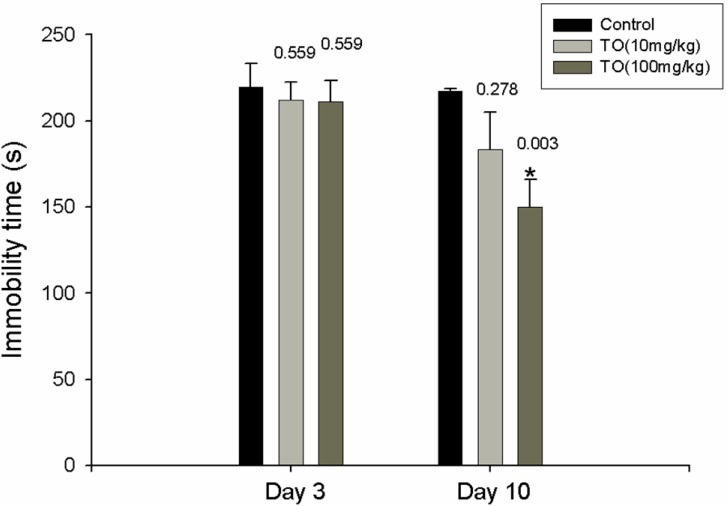
Effect of TO on immobility time in mice. One day after the first measurement of immobility, the administration of TO (10 and 100 mg/kg/day, p.o.) was started; this continued for a total of 10 days. Three days after the first administration, the second measurement of immobility was made. After the last administration (the 10th day), the third measurement of immobility was made. Values are the mean ± S.E. of twice experiments (n = 10). * *p* < 0.05 *vs.* distilled water-treated group.

We investigated the effect of TO by performing a FST. When placed into the cylinders for the first time, the mice swam around vigorously, apparently searching for an exit. After 2 to 3 min, their activity reduced and was replaced by periods of immobility of increasing duration, during which the mice remained floating passively in the water in a semi-horizontal position with their heads just above water level. After the first measurement of immobility time, the mice were divided into a control group and two TO groups on the basis of concentration (10 and 100 mg/kg) to match the swimming time in each group. The second measurement of immobility was performed 3 days after treatment with distilled water or TO. The immobility time after 3 days was not affected by TO treatment. After 10 days, the immobility time for the TO-treated group was lesser than that for the distilled water-treated group. In particular, the group treated with 100 mg/kg TO showed significantly lesser immobility time than that shown by the distilled water-treated group (164 ± 7.8 s *vs.* 215 ± 0.7 s; Figure 1).

### 2.2. Effects of TO on Blood Biochemical Parameters

The effect of TO on energy sources was investigated by analyzing the levels of Glc and Alb in mouse serum. Glc levels in the group treated with 100 mg/kg TO were significantly higher than those in the distilled water-treated group, and Alb levels remained unchanged in the TO-treated group. Moreover, fatigue-related BUN and LDH levels in the TO-treated groups (10 and 100 mg/kg) were lower than the corresponding levels in the distilled water-treated group (Table 1). Compared to the distilled water-treated group, the TO-treated groups showed unchanged CK levels.

Swimming is known to induce biochemical changes in blood [18]. BUN, CK, LDH, Glc, and Alb are fatigue-related blood biochemical parameters. The BUN test is routinely used to evaluate renal function. When TO was administered orally to mice, the BUN level decreased. CK and LDH are known to be accurate indicators of muscle damage [12,13]. LDH is known to catalyze the interconversion of pyruvate and lactate. Therefore, the level of LDH increases immediately after exercise. Surprisingly, in the present study, the LDH level was decreased after TO treatment, whereas CK level remained unchanged. After the FST, Glc levels were significantly increased by the oral administration of TO. This result suggests that TO may act as an energy source. Therefore, our results demonstrate that the fatigue indicators investigated in the mice were influenced by TO treatment. However, further studies are necessary to support these findings and to clarify the detailed mechanisms involved in the immune enhancement-like properties of TO.

**Table 1:** Effect of TO on blood biochemical parameters.

TO Treatment ^a^	0 mg/kg	10 mg/kg	100 mg/kg
BUN (mg/dL)	23.98 ± 0.58	19.00 ± 0.62 * (0.007)	17.16 ± 2.15 * (0.001)
LDH (IU/L)	1625.75 ± 113.75	637.25 ± 70.32 * (0.000)	640.50 ± 91.16 * (0.000)
CK (IU/L)	607.75 ± 67.25	440.25 ± 78.23 (0.493)	438.66 ± 48.22 (0.562)
Glc (mg/dL)	121.33 ± 11.34	130.5 ± 4.97 (0.962)	138.75 ± 2.83 * (0.042)
Alb (g/dL)	2.9 ± 0.08	2.4 ± 0.11 (0.051)	2.8 ± 0.13 (0.994)

^a^ Distilled water and TO were orally administered to mice, and then the levels of BUN, LDH, CK, Glc, and Alb were measured in serum 10 days after the first administration. Each level was determined by an autoanalyzer. Each data value indicates the mean ± SEM. * *p* < 0.05 indicates significant difference from results of the distilled water-treated group.

### 2.3. Effect of TO on rIFN-γ- Induced TNF-α, IL-12p70 Productions and mRNA Expressions in Mouse Peritoneal Macrophages

Macrophages are associated with many different processes, such as tissue remodeling during embryogenesis, wound repair, removal of damaged or senescent cells subsequent to injury or infection, hematopoiesis, and homeostasis. Another important role of macrophages is to protect against microbial invasion and to recognize and kill tumor cells. To demonstrate the immune-enhancing effect of TO, we also performed in vitro tests in mouse peritoneal macrophages. Macrophages produce cytokines such as IL-12 and TNF-α, which promote cell-mediated immunity [10]. In this study, TNF-α production and mRNA expression were increased by TO in combination with rIFN-γ treatment in mouse peritoneal macrophages. In addition, IL-12p70 production and mRNA expression were increased by TO in combination with rIFN-γ.

**Figure 2:**
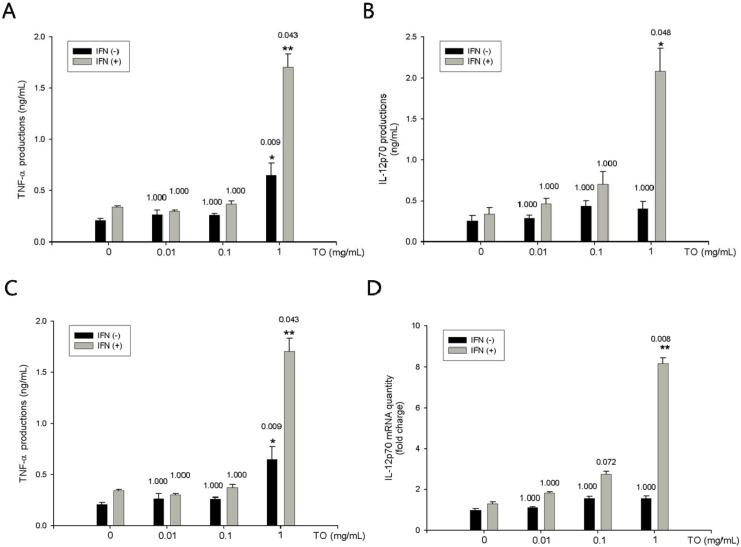
Effects of TO on rIFN-γ plus TO-induced TNF-α and IL-12p70 productions and mRNA expressions in peritoneal macrophages. (**A**) Peritoneal macrophages (3 × 10^5^ cells/well) were incubated for 1 h with rIFN-γ (10 U/mL). The peritoneal macrophages were then stimulated with TO (0.01–1 mg/mL) for 24 h. The supernatants were analyzed for cytokines expression by ELISA as described in the method. (B) Peritoneal macrophages (3 × 10^5^ cells/well) were incubated for 1 h with rIFN-γ (10 U/mL). The peritoneal macrophages were then stimulated with TO (0.01–1 mg/mL) for 6 h. Total RNA was prepared for real time RT-PCR analysis TNF-α and IL-12p70 expressions in peritoneal macrophages. The experiment was repeated three times and similar results were obtained. Values represent means ± S.E. of three independent experiments. * *p* < 0.05 *vs.* rIFN-γ (10 U/mL); ** *p* < 0.01 *vs.* rIFN-γ (10 U/mL) significant differences between treated groups were determined by ANOVA and Dunnett’s post-hoc test.

To determine the effect of TO on the production of TNF-αin mouse peritoneal macrophages, both non-primed (resting) and rIFN-γ-primed cells were treated with TO. First, we investigated cell viability to establish appropriate doses of TO for use in peritoneal macrophages. TO showed no cytotoxicity (≤1 mg/mL) in murine peritoneal macrophages (data not shown). Therefore, we used TO at concentrations of 0.01, 0.1, and 1 mg/mL *in vitro*. TNF-α production was measured in cell culture supernatants by using enzyme-linked immunosorbent assay (ELISA). As shown in Figure 2A, rIFN-γ-induced TNF-α production in the group treated with 1 mg/mL TO was significantly higher than that in the rIFN-γ-alone group. Assay performed using E-TOXATE kit (Sigma) revealed the TO extract to be free from endotoxins. The effect of TO on rIFN-γ-induced IL-12p70 production was investigated. Mouse peritoneal macrophages secreted very low levels of IL-12p70 after 24-h incubation with medium alone or rIFN-γ alone. However, TO (1 mg/mL) in combination with rIFN-γ or TO alone increased the production of IL-12p70 (Figure 2B). Real-time RT-PCR was performed to investigate whether TO increases the rIFN-γ-mediated induction of TNF-α and IL-12p70 expression at the transcriptional level. Treatment with TO was found to significantly increase the mRNA levels of TNF-α and IL-12p70 (Figure 2C, D), whereas it did not affect the expression of the housekeeping β-actin gene. 

### 2.4. Effect of TO on rIFN-γ- Induced IL-10 Production in Mouse Peritoneal Macrophages

**Figure 3:**
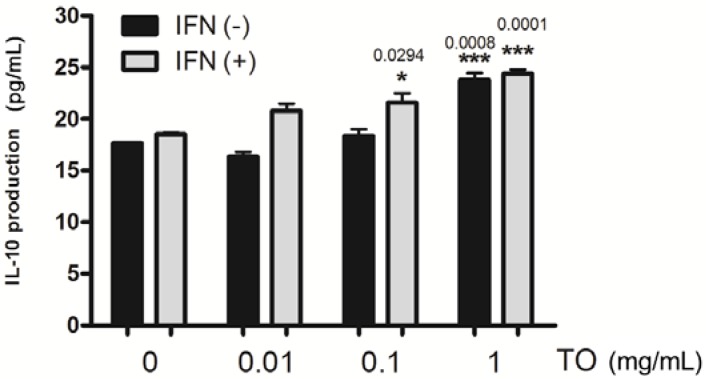
Effects of TO on rIFN-γ plus TO-induced IL-10 productions in peritoneal macrophages. Peritoneal macrophages (3 × 10^5^ cells/well) were incubated for 1 h with rIFN-γ (10 U/mL). The peritoneal macrophages were then stimulated with TO (0.01–1 mg/mL) for 24 h. The supernatants were analyzed for cytokines expression by ELISA as described in the method. The experiment was repeated three times and similar results were obtained. Values represent means ± S.E. of three independent experiments. * *p* < 0.05 *vs.* rIFN-γ (10 U/mL); *** *p* < 0.01 *vs.* rIFN-γ (10 U/mL) significant differences between treated groups were determined by ANOVA and Dunnett’s post-hoc test.

In previous reports, TO was shown to have anti-inflammatory effects [4,7]. Therefore, we determined the production of the pleiotropic cytokine IL-10 in TO-treated macrophages. To determine the effect of TO on the production of the pleiotropic cytokine IL-10 by mouse peritoneal macrophages, non-primed (resting) and rIFN-γ-primed cells were treated with TO. Similar to the production of the other cytokines, IL-10 production was measured in cell culture supernatants by using ELISA. As shown in Figure 3, compared to the rIFN-γ-alone group, the group treated with 1 mg/mL TO showed significantly higher rIFN-γ-induced IL-10 production level. It indicates that TO has a co-stimulatory effect in mouse peritoneal macrophages. TO treatment may enable the induction of cell-mediated immunity and limit inflammatory reactions in a severe inflammatory state. We suggest that IL-10 is a key cytokine in the TO-mediated regulation of immune function in murine macrophages.

### 2.5. Effects of TO on NO Production and iNOS Protein and mRNA Expressions

**Figure 4:**
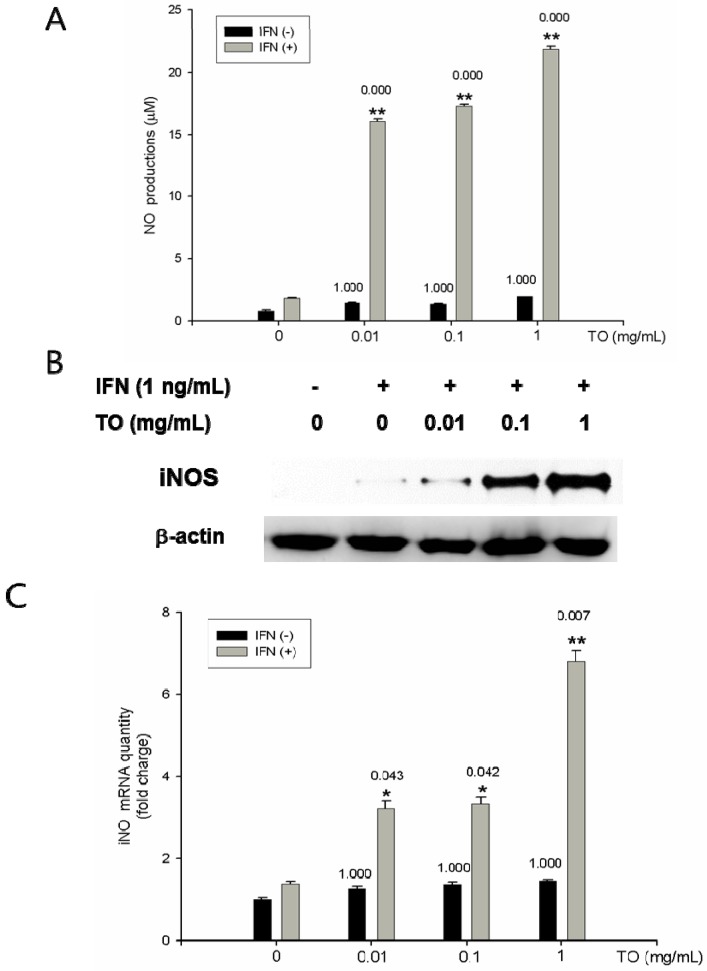
Effects of TO on NO productions, protein, and mRNA expressions in peritoneal macrophages. A) Peritoneal macrophages (3 × 10^5^ cells/well) were incubated for 1 h with rIFN-γ (10 U/mL). The peritoneal macrophages were then stimulated with TO (0.01–1 mg/mL) for 48 h. The supernatants were analyzed for NO production by Griess method as described in the method. B) Cells were pretreated with different concentrations (0.01–1 mg/mL) of TO for 12 h. Total cellular proteins (40 μg) were resolved by SDS-PAGE, transferred to nitrocellulose membranes, and detected with specific antibodies, as described in Materials and Methods. A representative immunoblot of three separate experiments is shown. C) Total RNA was prepared for real time RT-PCR analysis iNOS expressions in peritoneal macrophages. The experiment was repeated three times and similar results were obtained. Values represent means ± S.E. of three independent experiments. * *p* < 0.05 *vs.* rIFN-γ (10 U/mL); ** *p* < 0.01 *vs.* rIFN-γ (10 U/mL) significant differences between treated groups were determined by ANOVA and Dunnett’s post-hoc test.

To investigate the effect of TO on rIFN-γ-induced NO production, rIFN-γ-primed macrophages were co-treated with TO (0.01–1 mg/mL) for 48 h. Treatment with TO increased the production of NO in a dose-dependent manner (Figure 4A). To determine the effect of TO on rIFN-γ-induced iNOS expression in peritoneal macrophages, Western blotting was performed. As shown in Figure 4B, treatment with rIFN-γ significantly increased iNOS expression. Using real-time RT-PCR, we investigated the effect on the expression of iNOS mRNA. The treatment of mouse primary macrophages with TO (0.01–1 mg/mL) was found to significantly induce iNOS mRNA expression in a dose-dependent manner (Figure 4C). 

In co-cultures of macrophages and lymphoma cells, NO generated from macrophages has been shown to inhibit cellular respiration in the target cells [19]. Moreover, NO derived from Kupffer, natural killer (NK), and endothelial cells also contributes to tumoricidal activity against many types of tumors [20]. These studies suggest that NO has cytostatic and/or cytotoxic effects on tumor cells. In this study, we demonstrated that TO-induced NO production in mouse peritoneal macrophages could be highly stimulated by administration of rIFN-γ. Accordingly, iNOS expression was also increased by treatment with TO in combination with rIFN-γ. These results suggest that TO may provide a second signal for the synergistic induction of NO production in mouse peritoneal macrophages. Thus, NO production via iNOS activation indicates the various effects of TO, including antimicrobial, antitumor, and antiviral effects under specific conditions *in vivo*. In future experiments the effect of TO in primary cultured peritoneal macrophages from *in vivo*-treated mice will be investigated. This ex vivo test will be a clearer method to investigate the effect of TO on immunity in mice.

## 3. Experimental

### 3.1. Reagents

Thioglycollate (TG) was purchased from Difco Laboratories (Detroit, MI, USA). Dulbecco’s Modified Eagle’s Medium (DMEM), ampicillin, streptomycin and fetal bovine serum (FBS) were purchased from Gibco BRL (Grand Island, NY, USA). Murine recombinant interferon-γ (rIFN-γ) (1 × 10^7^ U/mL) was purchased from R&D Systems (Minneapolis, MI, USA). Anti-mouse TNF-α, anti-mouse IL-12, biotinylated anti-mouse TNF-α, anti-mouse IL-12, and recombinant mouse TNF-α, recombinant mouse IL-12 were purchased from Pharmingen (San Diego, CA, USA). Avidin-peroxidase, and 2'-azino-bis(3-ethylbenzithiazoline-6-sulfonic acid) (ABTS) substrate tablets were purchased from Sigma (St. Louis, MO, USA).

### 3.2. Preparation of TO

The plant sample was obtained from an oriental drug store, Daehak Pharmacy (Iksan, Jeonbuk, Korea), and classified and identified by local experts. Extract of TO was prepared by decocting the dried prescription of herbs with boiling distilled water. The duration of decoction was about 3 h. The decoction was filtered, lyophilized, and kept at 4 °C. Dilutions were made in distilled water then filtered through a 0.45 μm syringe filter.

### 3.3. Animals

The original stock of male ICR mice (4 weeks) were purchased from from Daehan Biolink (DaeJeon, South Korea), and the animals were maintained in the College of Oriental Medicine, Kyunghee University. The mice were housed 5–10 per cage in a laminar air-flow room maintained at a temperature of 22 ± 1 °C and relative humidity of 55 ± 10% throughout the study. Mice were treated in accordance with the current law and the NIH Guide for the Care and Use of Laboratory Animals.

### 3.4. FST

During 6-min FST, the duration of immobility was measured as previously described [21]. The apparatus consisted of two Plexiglas cylinders (height: 25 cm, diameter: 10 cm) placed side by side in Makrolon cage filled with water (10 cm height) at 23–25 °C. Two mice were tested simultaneously for a 6 min period inside the cylinders; a nontransparent screen placed between the two cylinders prevented the mice from seeing each other. The duration of immobility, after a delay of 2 min, was measured during a period of 4 min [21]. Each mouse was considered to be immobile when it ceased struggling and remained floating motionless in the water, making only those movements necessary to keep its head above water.

### 3.5. Preparation and Ingredient Analysis of Blood Serum

Changes in several blood biochemical parameters in the mice were measured after the FST. Blood samples were collected by cardiac puncture under ether anesthesia after the last FST. Then, serum was prepared by centrifugation at 3,000 rpm at 4 °C for 10 min. Contents of BUN, LDH, CK, Glc, and Alb were determined by the autoanalyzer (Hitachi 747, Hitachi, Tokyo, Japan). 

### 3.6. Peritoneal Macrophage Culture

TG-elicited macrophages were harvested 3 days after an intraperitoneal injection of 2.5 mL TG to mice, and isolated, as reported previously [22]. Using 8 mL of HBSS, which contained 10 U/mL heparin, performed peritoneal lavage was performed. Then, the cells were distributed in DMEM, which was supplemented with 10% heat-inactivated FBS, in 24-well tissue culture plates (3 × 10^5^ cells/well) which were incubated for 4 h at 37 °C in an atmosphere of 5% CO_2_. These were then washed three times with HBSS to remove non-adherent cells, and equilibrated with DMEM that contained 10% FBS before treatment.

### 3.7. Assay for Endotoxin Determination

The TO extract used in this experiment was found to be free from endotoxins, as determined within the limits of the E-TOXATE assay kit (Sigma), which was preformed according to manufacturer’s protocol. In this assay, saturation occurred at 40 EU/mL and the resolution limit was > 0.1 EU/mL. 

### 3.8. Cytokine Assays

Cytokine assays were performed by a modified ELISA as described previously [23]. The ELISA was performed by coating 96-well plates with mouse monoclonal Ab specific IL-12p70 and TNF-α. The plates were washed in PBS containing 0.05% Tween-20 (Sigma) and blocked with PBS containing 1% bovine serum albumin (BSA), 5% sucrose, and 0.05% NaN3 for 1 h. After additional washing, the sample or TNF-α, IL-12p70, and IL-10 standards was added and incubated at 37 °C for 2 h. After 2 h incubation at 37 °C the wells were washed and then 0.2 mg/mL each of biotinylated anti-mouse TNF-α, IL-12p70, and IL-10 were added and again incubated at 37 °C for 2 h. After washing the wells, avidin-peroxidase was added and the plates were incubated for 30 min at 37 °C. Wells were again washed and ABTS substrate was added. Color development was measured at 405 nm using an automated microplate ELISA reader. A standard curve was run on each assay plate using recombinant TNF-α, IL-12p70, and IL-10 in serial dilutions.

### 3.9. RNA Isolation and Real Time RT-PCR Analysis

Total RNA was prepared using the Easy-blue reagent (Intronbio, Korea), according to the manufacturer’s instructions. Total RNA (2.5 μg) was reverse transcribed into first-strand cDNA (Amersham Pharmacia Biotech, Oakville, ON, Canada) following the manufacturer’s procedure. The synthesised cDNA was used as a template for polymerase chain reaction (PCR) amplification. Real-time PCR was performed using Thermal Cycler Dice Real Time PCR System (Takara, Japan). The primers used for SYBR Green real-time RT-PCR were as follows: for TNF-α, sense primer, 5'-CACAGAAAGCATGATCCGCGACGT-3', and antisense primer, 5'-CGGCAGAGAGGAGGTTG ACTTTCT-3'; for IL-12p70, sense primer, 5'-GGAAGCACGGCAGCAGAATA-3' and antisense primer, 5'-AACTTGAGGGAGAAGTAGGAATGG-3'; for GAPDH, sense primer, 5'-GAGTCAAC GGATTTGGTCGT-3' and antisense primer, 5'-TTGATTTTGGAGGGATCTCG-3'. A dissociation curve analysis of TNF-α, IL-12p70, and GAPDH showed a single peak. PCRs were carried out for 40 cycles using the following conditions: denaturation at 95 °C for 5 s, annealing at 57 °C for 10 s, and elongation at 72 °C for 20 s. Mean Ct of the gene of interest was calculated from triplicate measurements and normalised with the mean Ct of a control gene, GAPDH.

### 3.10. Measurement of Nitrite Concentration

Peritoneal macrophages (3 × 10^5^ cells/well) were cultured with rIFN-γ (10 U/mL) for 6 h. The cells were then stimulated with various concentrations of TO. NO synthesis in cell cultures was measured by a microplate assay method as previously described [24]. To measure nitrite, 100 μL aliquots were removed from conditioned medium and incubated with an equal volume of Griess reagent (1% sulphanilamide/0.1% *N*-(1-naphtyl)-ethylenediamine dihydrochloride/2.5% H_3_PO_4_) at room temperature for 10 min. The absorbance at 540 nm was determined by an automatic microplate reader (Molecular Devices Corp., Sunnyvale, CA, USA). NO^2−^ was determined by using sodium nitrite as a standard. The cell-free medium alone contained 5–8 μM of NO^2−^. This value was determined in each experiment and subtracted from the value obtained from the medium with peritoneal macrophages.

### 3.11. Western Blot Analysis

Peritoneal macrophages (5 × 10^6^ cells/well) were incubated for 6 h with rIFN-γ (10 U/mL). The cells were then stimulated with TO (0.01–1 mg/mL) for 12 h. Whole cell lysates were made by boiling peritoneal macrophages in sample buffer (62.5 mM Tris-HCl, pH 6.8, 2% sodium dodecyl sulfate (SDS), 20% glycerol, and 10% 2-mercaptoethanol). Proteins in the cell lysates were then separated by 10% SDS-polyacrylamide gel electrophoresis and transferred to nitrocellulose paper. The membrane was then blocked with 5% skim milk in PBS containing 0.05% Tween-20 for 1 h at room temperature and then incubated with anti-iNOS antibodies. After washing in PBS-Tween-20 three times, the blot was incubated with secondary antibody for 30 min and the antibody-specific proteins were visualized by the enhanced chemiluminesence detection system according to the recommended procedure (Amersham Corp., Newark, NJ, USA).

### 3.12. Statistical Analysis

The experiments shown are a summary of the data from at least-three experiments and are expressed as the mean ± S.E. of independent experiments, and statistical analyses were performed by one-way analysis of variance (ANOVA) with Tukey, and Duncan post hoc test to express the difference among the groups. All statistical analyses were performed using SPSS v12.0 statistical analysis software. A value of *p* < 0.05 was considered to indicate statistical significance.

## 4. Conclusions

In conclusion, our results showed that TO decreased immobility time in the FST model and resulted in changes in the levels of fatigue-related metabolites. Furthermore, TO increased TNF-α, IL-12p70, and IL-10 levels, and NO production in primary cultured peritoneal macrophages. Our study provides new insights into the pharmacological actions of TO as a potential treatment with immune-enhancing effects. 
